# The effect of protein timing on muscle strength and hypertrophy: a meta-analysis

**DOI:** 10.1186/1550-2783-10-53

**Published:** 2013-12-03

**Authors:** Brad Jon Schoenfeld, Alan Albert Aragon, James W Krieger

**Affiliations:** 1 Department of Health Science, Lehman College, Bronx, NY, USA; 2 California State University, Northridge, CA, USA; 3 Weightology, LLC, Issaquah, WA, USA

## Abstract

Protein timing is a popular dietary strategy designed to optimize the adaptive response to exercise. The strategy involves consuming protein in and around a training session in an effort to facilitate muscular repair and remodeling, and thereby enhance post-exercise strength- and hypertrophy-related adaptations. Despite the apparent biological plausibility of the strategy, however, the effectiveness of protein timing in chronic training studies has been decidedly mixed. The purpose of this paper therefore was to conduct a multi-level meta-regression of randomized controlled trials to determine whether protein timing is a viable strategy for enhancing post-exercise muscular adaptations. The strength analysis comprised 478 subjects and 96 ESs, nested within 41 treatment or control groups and 20 studies. The hypertrophy analysis comprised 525 subjects and 132 ESs, nested with 47 treatment or control groups and 23 studies. A simple pooled analysis of protein timing without controlling for covariates showed a small to moderate effect on muscle hypertrophy with no significant effect found on muscle strength. In the full meta-regression model controlling for all covariates, however, no significant differences were found between treatment and control for strength or hypertrophy. The reduced model was not significantly different from the full model for either strength or hypertrophy. With respect to hypertrophy, total protein intake was the strongest predictor of ES magnitude. These results refute the commonly held belief that the timing of protein intake in and around a training session is critical to muscular adaptations and indicate that consuming adequate protein in combination with resistance exercise is the key factor for maximizing muscle protein accretion.

## Background

Protein timing is a popular dietary strategy designed to optimize the adaptive response to exercise [1]. The strategy involves consuming protein in and around a training session in an effort to facilitate muscular repair and remodeling, and thereby enhance post-exercise strength- and hypertrophy-related adaptations [2]. It is generally accepted that protein should be consumed just before and/or immediately following a training session to take maximum advantage of a limited anabolic window [3]. Proponents of the strategy claim that, when properly executed, precise intake of protein in the peri-workout period can augment increases in fat-free mass [4]. Some researchers have even put forth the notion that the timing of food intake may have a greater positive effect on body composition than absolute daily nutrient consumption [5].

A number of studies support the superiority of protein timing for stimulating increases in acute protein synthesis pursuant to resistance training when compared to placebo [6-9]. Protein is deemed to be the critical nutrient required for optimizing post-exercise protein synthesis. The essential amino acids, in particular, are believed primarily responsible for enhancing this response, with little to no contribution seen from provision of non-essential amino acids [10,11]. Borsheim et al. [10] found that a 6 g dose of essential amino acids (EAAs) consumed immediately post-exercise produced an approximate twofold increase in net protein balance compared to a comparable dose containing an approximately equal mixture of essential and non-essential amino acids, indicating a dose–response relationship up to 6 g EAAs. However, increasing EAA intake beyond this amount has not been shown to significantly heighten post-exercise protein synthesis [2]. There is limited evidence that carbohydrate has an additive effect on enhancing post-exercise muscle protein synthesis when combined with amino acid ingestion [12], with a majority of studies failing to demonstrate any such benefit [13-15].

Despite the apparent biological plausibility of the strategy, the effectiveness of protein timing in chronic training studies has been decidedly mixed. While some studies have shown that consumption of protein in the peri-workout period promotes increases in muscle strength and/or hypertrophy [16-19], others have not [20-22]. In a review of literature, Aragon and Schoenfeld [23] concluded that there is a lack of evidence to support a narrow “anabolic window of opportunity” whereby protein need to be consumed in immediate proximity to the exercise bout to maximize muscular adaptations. However, these conclusions were at least in part a reflection of methodological issues in the current research. One issue in particular is that studies to date have employed small sample sizes. Thus, it is possible that null findings may be attributable to these studies being underpowered, resulting in a type II error. In addition, various confounders including the amount of EAA supplementation, matching of protein intake, training status, and variations in age and gender between studies make it difficult to draw definitive conclusions on the topic. Thus, by increasing statistical power and controlling for confounding variables, a meta-analysis may help to provide clarity as to whether protein timing confers potential benefits in post-exercise skeletal muscle adaptations.

A recent meta-analysis by Cermak et al. [24] found that protein supplementation, when combined with regimented resistance training, enhances gains in strength and muscle mass in both young and elderly adults. However, this analysis did not specifically investigate protein timing per se. Rather, inclusion criteria encompassed all resistance training studies in which at least one group consumed a protein supplement or modified higher protein diet. The purpose of this paper therefore is to conduct a meta-analysis to determine whether timing protein near the resistance training bout is a viable strategy for enhancing muscular adaptations.

## Methodology

### Inclusion criteria

Only randomized controlled trials or randomized crossover trials involving protein timing were considered for inclusion. Protein timing was defined here as a study where at least one treatment group consumed a minimum of 6 g essential amino acids (EAAs) ≤ 1 hour pre- and/or post-resistance exercise and at least one control group did not consume protein < 2 hours pre- and/or post-resistance exercise. Resistance training protocols had to span at least 6 weeks and directly measure dynamic muscle strength and/or hypertrophy as a primary outcome variable. There were no restrictions for age, gender, training status, or matching of protein intake, but these variables were controlled via subgroup analysis using meta-regression.

### Search strategy

To carry out this review, English-language literature searches of the PubMed and Google Scholar databases were conducted for all time periods up to March 2013. Combinations of the following keywords were used as search terms: “nutrient timing”; “protein supplementation”; “nutritional supplementation”; “protein supplement”; “nutritional supplement”; “resistance exercise”; “resistance training”; “strength training”. Consistent with methods outlined by Greenhalgh and Peacock [25], the reference lists of articles retrieved in the search were then screened for any additional articles that had relevance to the topic. Abstracts from conferences, reviews, and unpublished dissertations/theses were excluded from analysis. A total of 34 studies were identified as potentially relevant to this review. To reduce the potential for selection bias, each of these studies were independently perused by two of the investigators (BJS and AAA), and a mutual decision was made as to whether or not they met basic inclusion criteria. Study quality was then assessed with the PEDro scale, which has been shown to be a valid measure of the methodologic quality of RCTs [26] and possesses acceptable inter-rater reliability [27]. Only those studies scoring ≥5 on the PEDro scale--a value considered to be of moderate to high quality [27]-were accepted for analysis. Any inter-reviewer disagreements were settled by consensus and/or consultation with the third investigator. Initial pre-screening revealed 29 potential studies that investigated nutrient timing with respect to muscular adaptations. Of these studies, 3 did not meet criteria for sufficient supplemental protein intake [28-30] and in another the timing of consumption was outside the defined post-workout range [31]. Thus, a total of 25 studies ultimately were deemed suitable for inclusion. Two of the studies were subsequently excluded because they did not contain sufficient data for calculating an effect size and attempts to obtain this information from the authors were unsuccessful [19,32], leaving a total 23 studies suitable for analysis. The average PEDro score of these studies was 8.7, indicating an overall high level of methodological quality. Table 1 summarizes the studies meeting inclusion criteria.

**Table 1:** Summary of studies meeting inclusion criteria

**Study**	**Subjects**	**Supplementation**	**Protein matched with control?**	**Anthropometric and/or body composition assessment method**	**Training protocol**	**Strength results**	**Body composition results**
Antonio et al., [33]	19 untrained young women	18.3 g EAA or an equal dose of cellullose placebo taken (collectively) 20 minutes pre and post-exercise	No	DXA	Periodized progressive resistance training consisting of exercises for all major muscle groups performed 3 days/wk for 6 wks	Total weight lifted at the 12 RM intensity did not significantly change in either group	No significant body composition changes occurred in either group
Goddard et al., [34]	17 untrained older men (60–80 y)	12 g of essential amino acids and 72 g (total) of fructose and dextrose consumed immediately after exercise	No	Computed tomography (CT).	Progressive resistance training consisting of knee extensions preformed 3 days/wk for 12 wks	Training produced a significant increase in 1RM strength and measures of maximal torque, no differences between groups	No significant differences in muscle CSA increase between groups
Rankin et al., [35]	13 untrained young men	Chocolate milk (providing a protein dose of 0.21 g/kg) or a CHO-electrolyte beverage (Gatorade) immediately after exercise	No	Dual X-ray absorptiometry (DXA) and multiple upper & lower body circumference measurements	Periodized progressive resistance training consisting of exercises for all major muscle groups performed 3 days/wk for 10 wks	1 RM strength increased in all exercises, with no significant difference between groups	No significant differences in fat reduction, mean mass gain, or circumference changes between groups
Andersen et al., [36]	22 untrained young men	25 g protein (combination of whey, casein, egg white, and glutamine) or 25 g maltodextrin immediately before and after exercise	No	Muscle biopsy	Periodized progressive resistance training consisting of lower body exercises performed 3 days/wk for 14 wks	Squat jump height increased only in the protein group, whereas countermovement jump height and peak torque during slow isokinetic muscle contraction increased similarly in both groups.	The protein group showed hypertrophy of type I & II muscle fibers, whereas no significant change occurred in the CHO group
Bird et al., [37]	32 untrained young men	6 g EAA or 6% CHO solution + 6 g EAA or placebo during exercise	No	DXA and muscle biopsy	Progressive resistance training consisting of exercises for all major muscle groups performed 2 days/wk for 12 wks	Training caused a significant increase in 1RM in the leg press similarly in both treatment groups compared to placebo, isokinetic strength increased in all groups, with no differences between groups	CHO + EAA showed greater gains in fat-free mass compared to placebo, fat mass decreased in all groups without any significant difference between groups
Coburn et al., [38]	33 untrained young men	20 g whey + 6.2 g leucine or 26.2 g maltodextrin 30 minutes prior to and immediately after exercise	No	Magnetic resonance imaging (MRI)	Progressive resistance training consisting of knee extensions performed 3 days/wk for 8 wks	Significantly greater 1 RM strength increase in the trained limb in the protein group compared to placebo	No significant body composition changes occurred in any of the groups, CSA increases did not differ between the protein and placebo groups
Candow, Burke, et al., [39]	27 untrained young men & women	Whey (1.2 g/kg) + sucrose (0.3 g/kg) or placebo (1.2 g/kg maltodextrin + 0.3 g/kg sucrose)	No	DXA	Progressive, periodized resistance training consisting of exercises for all major muscle groups performed 4 days/wk for 6 wks	1 RM strength increases in the squat and bench press were significantly greater in the protein groups than placebo	Lean mass increase was significantly greater in the protein groups than placebo
		*Note that only the soy treatment was excluded from analysis.*					
Candow, Chilibeck, et al., [40]	29 untrained older men	Multi-ingredient supplement containing a protein dose of 0.3 g/kg immediately before exercise and a CHO-based placebo immediately after, or the reverse order of the latter, or placebo before & after exercise	No	Air-displacement plethysmography, ultrasound	Progressive resistance training consisting of exercises for all major muscle groups performed 3 days/wk for 12 wks	1 RM strength increases in the leg press & bench press occurred in all groups, no significant differences between groups	Lean mass and muscle thickness increased in all groups, no significant difference between groups
Cribb and Hayes, [16]	23 young recreational male bodybuilders	1 g/kg of a supplement containing 40 g whey isolate, 43 g glucose, and 7 g creatine monohydrate consumed either immediately before and after exercise or in the early morning and late evening	Yes	DXA and muscle biopsy	Progressive resistance training consisting of exercises for all major muscle groups performed 3 days/wk for 10 wks	Immediate pre-post supplementation caused greater increases in 1-RM in 2 out of 3 exercises	Significant increases in lean body mass and muscle CSA of type II fibers in immediate vs. delayed supplementation
Hartman et al., [41]	56 untrained young men	17.5 g protein within milk or a soy beverage, or CHO control immediately after exercise and again 1 hr after exercise	No	DXA and muscle biopsy	Progressive resistance training consisting of exercises for all major muscle groups performed 5 days/wk for 12 wks	All groups experienced 1RM strength gains, but no between-group differences were seen	Type II muscle fiber area increased in all groups, but with greater increases in the milk group than in the soy and control groups, fat-free mass increased to a greater extent in the milk group compared to the soy & control groups
		*Note that only the soy treatment was excluded from analysis.*					
Hoffman et al., [42]	21 well-trained young men	42 g protein within a multi-ingredient supplement or a CHO placebo taken once in the morning and again after training	No	DXA	Progressive, periodized resistance training consisting of exercises for all major muscle groups performed 4 days/wk for 12 wks	1 RM bench press strength (but not squat strength) significantly increased in the protein group, while no measures of strength increased in the placebo group	No significant between-group or absolute changes in body composition occurred
Willoughby et al., [17]	19 untrained young men	20 g whey-dominant protein or 20 g dextrose consumed 1 hour before and after exercise	No	Hydrostatic weighing, muscle biopsy, surface measurements	Progressive resistance training consisting of exercise for all major muscle groups performed 4 days/wk for 10 wks	Protein supplementation caused greater increases in relative strength (maximal strength corrected for bodyweight) in bench press & leg press	Significant increase in total body mass, fat-free mass, and thigh mass with protein vs. carb supplementation
Eliot et al., [43]	42 untrained older men	35 g whey protein + CHO-electrolyte solution, or whey/CHO + 5 g creatine, or creatine-only, or CHO placebo	No	DXA and bioelectrical impedance	Progressive resistance training consisting of exercise for all major muscle groups performed 3 days/wk for 14 wks	Not measured	No significant effects of any of the whey and/or creatine treatments were seen beyond body composition changes caused by training alone
		*Note that creatine treatments were excluded from analysis*					
Mielke et al., [44]	39 untrained young men	20 g whey protein + 6.2 g of leucine or 20 g maltodextrin 30 minutes before and immediately after exercise	No	Hydrodensitometry,	Dynamic constant external resistance (DCER) bilateral leg extension and bench press exercises were performed 3 days/wk for 8 wks.	1 RM strength increased significantly in both groups without any between-group differences	No significant training-induced changes in body composition in either group,
Verdijk et al., [21]	28 untrained elderly men	10 g casein hydrolysate or placebo consumed immediately before and after exercise	No	DXA, CT, and muscle biopsy	Progressive resistance training consisting leg press and knee extension performed 3 days/wk for 12 wks	1 RM leg press & leg extension strength increased, with no significant difference between groups	No significant differences in muscle CSA increase between groups
Hoffman et al., [20]	33 well-trained young men	Supplement containing 42 g protein (milk/collagen blend) and 2 g carbohydrate consumed either immediately before and after exercise or in the early morning and late evening	Yes	DXA	Progressive resistance training consisting exercises for the major muscle groups peformed 4 days/wk for 10 wks.	1 RM & 5 RM bench press & squat strength increased, with no significant difference between groups	No significant differences in total body mass or lean body mass between groups.
Hulmi et al., [18]	31 untrained young men	15 g whey isolate or placebo consumed immediately before and after exercise	No	MRI, muscle biopsy	Progressive, periodized total body resistance training consisting of exercises for all major muscle groups trained performed 2 days/wk for 21 wks	Strength increased similarly in the protein & placebo group, but only the protein group increased isometric leg extension strength vs the control group	Significant increase in CSA of the vastus lateralis but not of the other quadriceps muscles in the protein group vs placebo
Josse et al., [45]	20 untrained young women	18 g protein within milk or an isocaloric maltodextrin placebo immediately after exercise and again 1 hr later	No	DXA	Progressive, periodized resistance training consisting of exercises for all major muscle groups performed 5 days/wk for 12 wks	1 RM strength increased similarly in both groups, but milk significantly outperformed placebo in the bench press	Lean mass increased in both groups but to a significantly greater degree in the milk group, fat mass decreased in the milk group only
Walker et al., [46]	30 moderately trained men and women	19.7 g of whey protein and 6.2 g leucine or isocaloric CHO placebo 30–45 minutes before exercising and the second packet 30–45 minutes after exercising.	No	DXA	Bodyweight-based exercises and running at least 3 days/wk, externally loaded training not specified	1 RM bench press strength increased significantly in the protein group only	Total mass, fat-free mass, and lean body mass increased significantly in the protein group only
Vieillevoye et al., [47]	29 untrained young men	15 g EAA + 15 g saccharose. or 30 g saccharose consumed with breakfast and immediately after exercise	No	Ultrasonography, 3-site skinfold assessment with calipers, 3-site circumference measurements	Progressive, periodized resistance training consisting of exercises for all major muscle groups performed 2 days/wk for 12 wks	Maximal strength significantly increased in both groups, with no between-group diffrerence	Muscle mass significantly increased in both groups with no differences between groups, muscle thickness of the gastrocnemius medialis significantly increased in the EAA group only
Wycherly et al., [22]	34 untrained, older men & women w/type 2 diabetes	21 g protein, 0.7 g fat, 29.6 g carbohydrate consumed either immediately prior to, or at least 2 h following exercise	Yes	DXA, waist circumference	Progressive resistance training consisting of exercises for all major muscle groups performed 3 days/wk for 16 wks	Not measured	Fat mass, fat-free mass, and waist circumference decreased with no significant differences between groups
Erskine et al., [48]	33 untrained young men	20 g whey protein or placebo consumed immediately before and after exercise	No	MRI	4-6 sets of elbow flexion performed 3 days/wk for 12 weeks	No significant differences in maximal isometric voluntary force or 1 RM strength between groups	No significant differences in muscle CSA between groups
Weisgarber et al., [49]	17 untrained young men and women	Whey protein dosed at 0.3 g/kg or isocaloric CHO immediately before, during, and after exercise	No	DXA and ultrasound	Progressive resistance training consisting of exercises for all major muscle groups performed 4 days/wk for 8 wks	1 RM strength in the chest press increased in both groups without any between-group difference	Significant increases in muscle mass were seen without any difference between groups

### Coding of studies

Studies were read and individually coded by two of the investigators (BJS and AAA) for the following variables: Descriptive information of subjects by group including gender, body mass, training status (trained subjects were defined as those with at least one year resistance training experience), age, and stratified subject age (classified as either young [18–49 years] or elderly [50+ years]; whether or not total daily protein intake between groups was matched; whether the study was an RCT or crossover design; the number of subjects in each group; blinding (classified as single, double, or unblinded); duration of the study; type of hypertrophy measurement (MRI, CT, ultrasound, biopsy, etc.) and region/muscle of body measured, if applicable; lean body mass measurement (i.e. DXA, hydrostatic weighing, etc.), if applicable, and; strength exercise (s) employed for testing, if applicable. Coding was cross-checked between coders, and any discrepancies were resolved by mutual consensus. To assess potential coder drift, 5 studies were randomly selected for recoding as described by Cooper et al. [50]. Per case agreement was determined by dividing the number of variables coded the same by the total number of variables. Acceptance required a mean agreement of 0.90.

### Calculation of effect size

For each 1-RM strength or hypertrophy outcome, an effect size (ES) was calculated as the pretest-posttest change, divided by the pretest standard deviation (SD) [51]. The sampling variance for each ES was estimated according to Morris and DeShon [51]. Calculation of the sampling variance required an estimate of the population ES, and the pretest-posttest correlation for each individual ES. The population ES was estimated by calculating the mean ES across all studies and treatment groups [51]. The pretest-posttest correlation was calculated using the following formula [51]:

$$r=\left({s_{1}}^{2}+{s_{2}}^{2}-{s_{D}}^{2}\right)/\left(2\;s_{1}s_{2}\right)$$

where s_1_ and s_2_ are the SD for the pre- and posttest means, respectively, and s_D_ is the SD of the difference scores. Where s_2_ was not reported, s_1_ was used in its place. Where s_D_ was not reported, it was estimated using the following formula [52]:

$$s_{D}=\sqrt{\left(\left({s_{1}}^{2}/n\right)+\left({s_{2}}^{2}/n\right)\right)}$$

### Statistical analyses

Meta-analyses were performed using hierarchical linear mixed models, modeling the variation between studies as a random effect, the variation between treatment and control groups as a random effect nested within studies, and group-level predictors as fixed effects [53]. The within-group variances were assumed known. Observations were weighted by the inverse of the sampling variance [51]. An intercept-only model was created, estimating the weighted mean ES across all studies and treatment groups. Second, a basic model was created which only included the class of the group (treatment or control) as a predictor. A full model was then created with the following predictors: the class of the group (treatment or control), whether or not the groups were protein matched, training status (experienced or novice), blinding (double, single, or none), gender (male, female, or mixed), age (young or old), body mass in kg, and the duration of the study in weeks. The full model was then reduced by removing one predictor at a time, starting with the most insignificant predictor [54]. The final model represented the reduced model with the lowest Akaike’s Information Corrected Criterion (AICC) [55] and that was not significantly different (P > 0.05) from the full model when compared using a likelihood ratio test (LRT). Model parameters were estimated by the method of restricted maximum likelihood (REML) [56]; an exception was during the model reduction process, in which parameters were estimated by the method of maximum likelihood (ML), as LRTs cannot be used to compare nested models with REML estimates. Denominator df for statistical tests and CIs were calculated according to Berkey et al. [57]. The treatment/control classification variable was not removed during the model reduction process.

Separate analyses were performed for strength and hypertrophy. ESs for both changes in cross-sectional area (CSA) and FFM were pooled in the hypertrophy analysis. However, because resistance exercise is associated with the accretion of non-muscle tissue, separate sub-analyses on CSA and FFM were performed. Because the effect of protein timing might interact with whether the treatment and control groups were matched for total protein intake, an additional model was created that included an interaction term between the treatment/control classification variable and the protein match variable. Also, because the effect of protein timing might vary by training experience, a model was created that included an interaction term between the treatment/control classification variable and the training status variable. Adjustment for post hoc multiple comparisons was performed using a simulation-based procedure [58]. All analyses were performed using SAS Enterprise Guide Version 4.2 (Cary, NC). Effects were considered significant at P ≤ 0.05. Data are reported as means (±SEs) and 95% CIs.

## Results

### Study characteristics

The strength analysis comprised 478 subjects and 96 ESs, nested within 41 treatment or control groups and 20 studies. The weighted mean strength ES across all studies and groups was 1.39 ± 0.24 (CI: 0.88, 1.90). The hypertrophy analysis comprised 525 subjects and 132 ESs, nested with 47 treatment or control groups and 23 studies. The weighted mean hypertrophy ES across all studies and groups was 0.47 ± 0.08 (CI: 0.31, 0.63).

### Basic model

**Figure 1:**
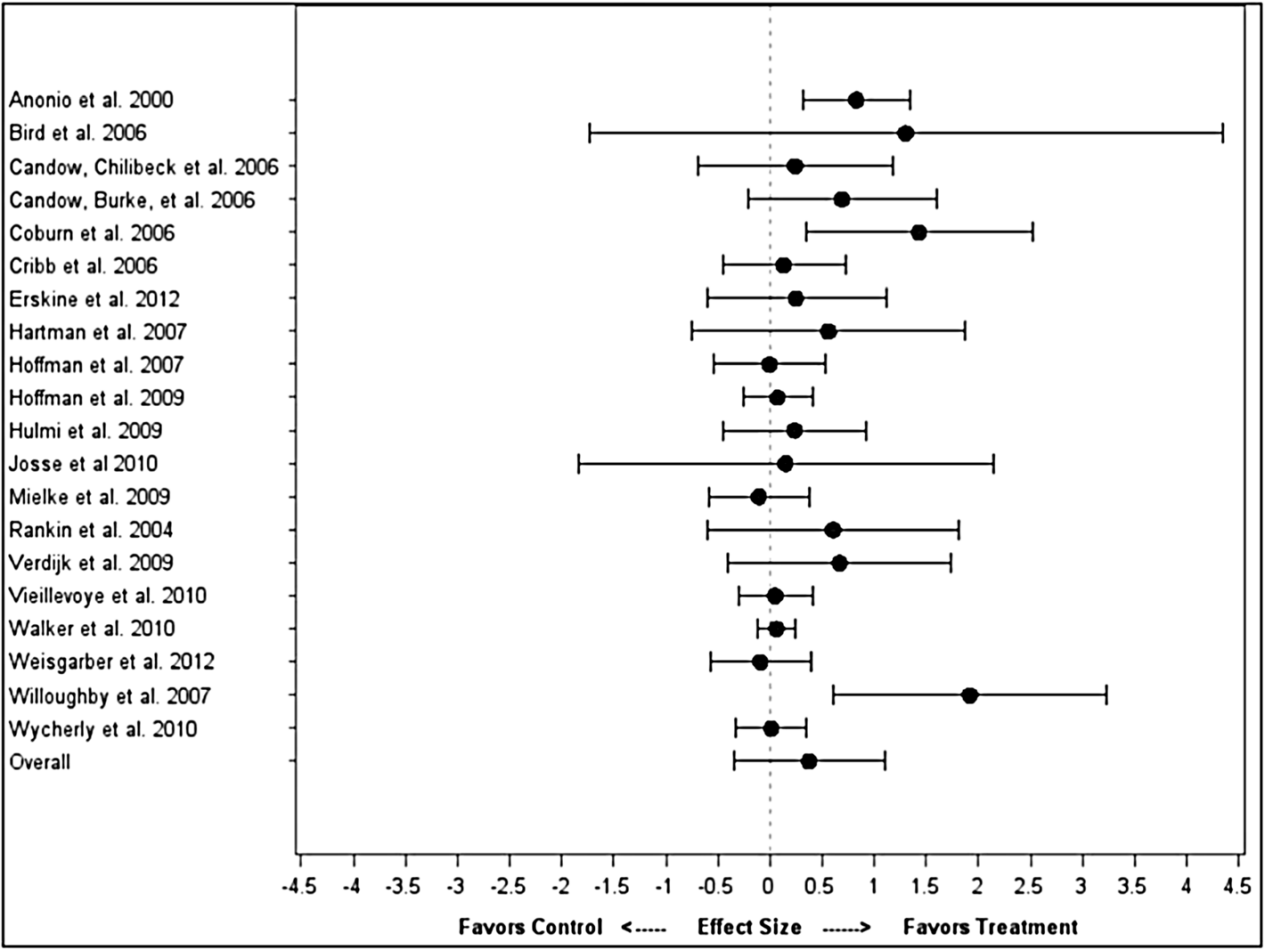
Impact of protein timing on strength by study.

**Figure 2:**
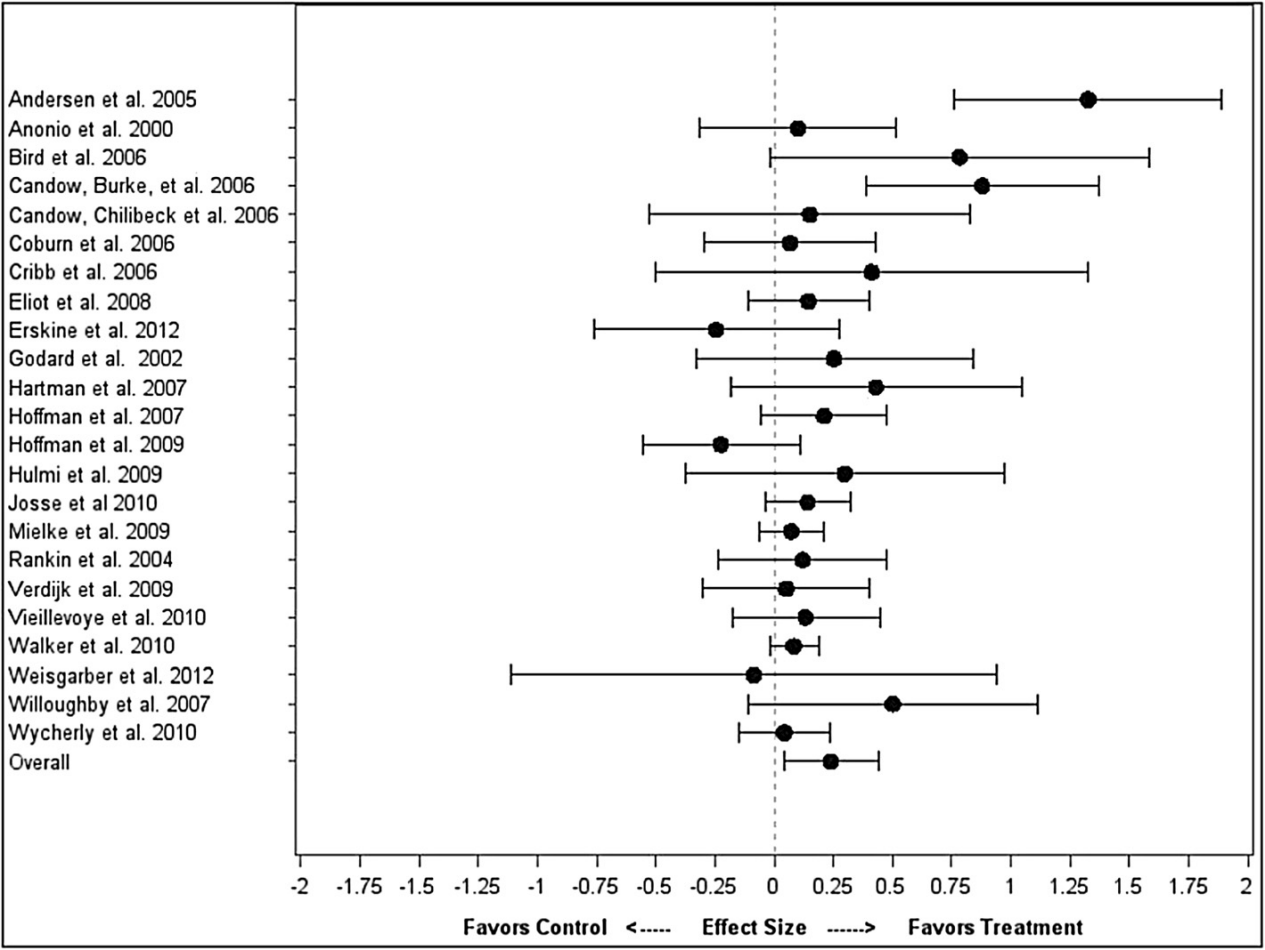
Impact of protein timing on hypertrophy by study.

There was no significant difference between the treatment and control for strength (difference = 0.38 ± 0.36; CI: -0.34, 1.10; P = 0.30). The mean strength ES difference between treatment and control for each individual study, along with the overall weighted mean difference across all studies, is shown in Figure 1. For hypertrophy, the mean ES was significantly greater in the treatment compared to the control (difference = 0.24 ± 0.10; CI: 0.04, 0.44; P = 0.02). The mean hypertrophy ES difference between treatment and control for each individual study, along with the overall weighted mean difference across all studies, is shown in Figure 2.

### Full model

In the full meta-regression model controlling for all covariates, there was no significant difference between the treatment and control for strength (difference = 0.28 ± 0.40; CI: -0.52, 1.07; P = 0.49) or hypertrophy (difference =0.16 ± 0.11; CI: -0.07, 0.38; P = 0.18).

### Reduced model: strength

After the model reduction procedure, only training status and blinding remained as significant covariates. The reduced model was not significantly different from the full model (P = 0.73). In the reduced model, there was no significant difference between the treatment and control (difference = 0.39 ± 0.36; CI: -0.34, 1.11; P = 0.29). The mean ES for control was 0.93 ± 0.31 (CI: 0.32, 1.54). The mean ES for treatment was 1.31 ± 0.30 (CI: 0.71, 1.92).

### Reduced model: hypertrophy

After the model reduction procedure, total protein intake, study duration, and blinding remained as significant covariates. The reduced model was not significantly different from the full model (P = 0.87). In the reduced model, there was no significant difference between the treatment and control (difference = 0.14 ± 0.11; CI: -0.07, 0.35; P = 0.20). The mean ES for control was 0.36 ± 0.09 (CI: 0.18, 0.53). The mean ES for treatment was 0.49 ± 0.08 (CI: 0.33, 0.66). Total protein intake (in g/kg) was the strongest predictor of ES magnitude (estimate = 0.41 ± 0.14; CI: 0.14, 0.69; P = 0.004).

**Figure 3:**
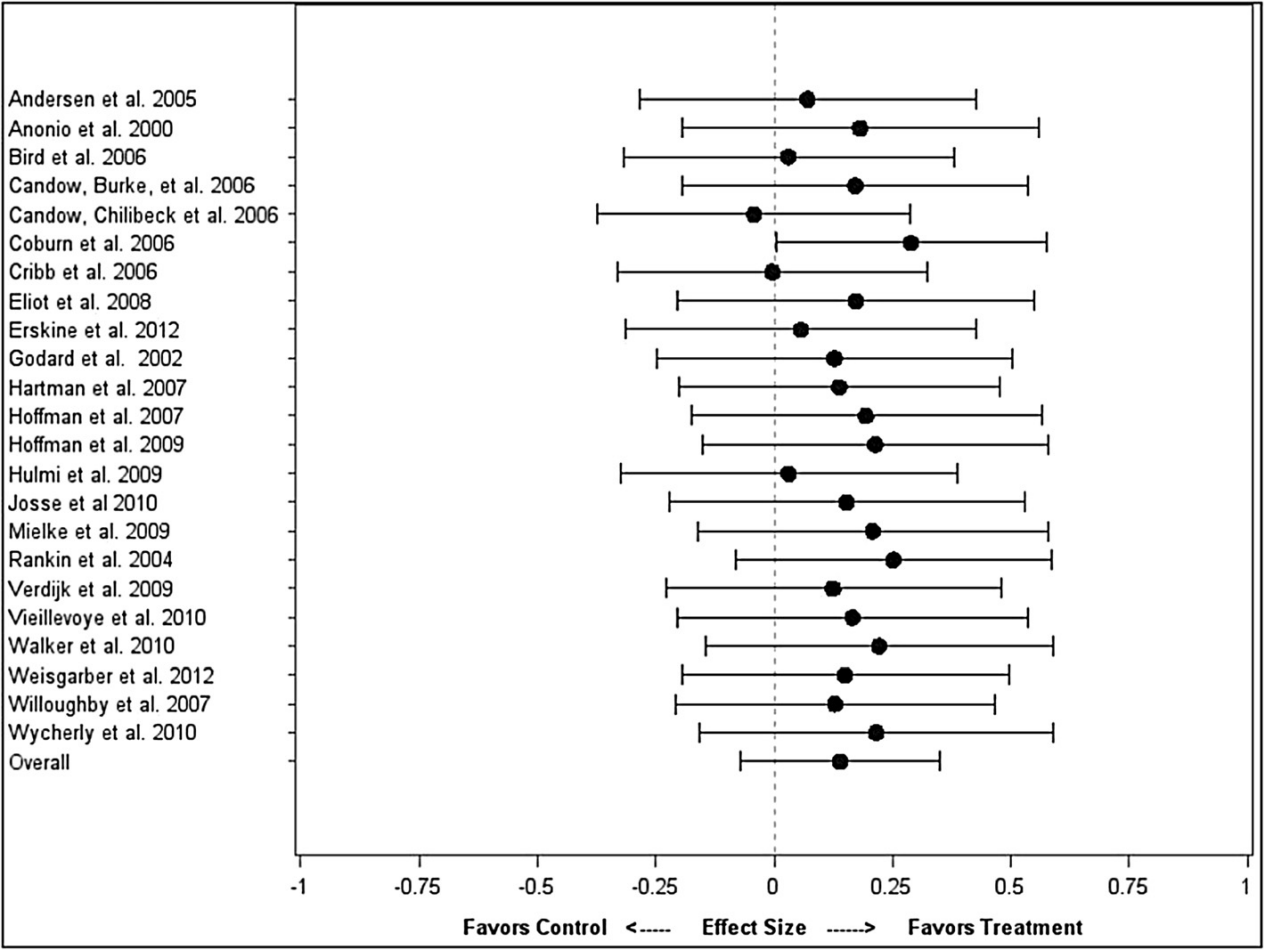
Impact of protein timing on hypertrophy by study, adjusted for total protein intake.

To confirm that total protein intake was mediator variable in the relationship between protein timing and hypertrophy, a model with only total protein intake as a covariate was created. The difference between treatment and control was not significant (difference = 0.14 ± 0.11; CI: -0.07, 0.35,; P = 0.19). Total protein intake was a significant predictor of ES magnitude (estimate = 0.39 ± 0.15; CI: 0.08, 0.69; P = 0.01). Figure 3 shows the total protein intake-adjusted ES’s for each study, as well as the overall effect from the meta-regression with only total protein intake as a covariate.

### Interactions

For strength, the interaction between treatment and training status was nearly significant (P = 0.051), but post hoc comparisons between treatment and control within each training status classification were not significant (adjusted P = 0.47 for difference within non-experienced groups, and adjusted P = 0.99 for difference within experienced groups). There was no significant interaction between treatment and whether groups were protein matched (P = 0.43). For hypertrophy, there was no significant interaction between treatment and training status (P = 0.63) or treatment and protein matching (P = 0.59).

### Hypertrophy sub-analyses

Separating the hypertrophy analysis into CSA or FFM did not materially alter the outcomes. For FFM, there was no significant difference between treatment and control (difference = 0.08 ± 0.07; CI: -0.07, 0.24; P = 0.27). Total protein intake remained a strong predictor of ES magnitude (estimate = 0.39 ± 0.07; CI: 0.25, 0.53; P < 0.001). For CSA, there was no significant difference between treatment and control (difference = 0.14 ± 0.16; CI: -0.17, 0.46; P = 0.37). Total protein intake was again a predictor of ES magnitude (estimate = 0.55 ± 0.24; CI: 0.08, 1.20; P = 0.02).

## Discussion

This is the first meta-analysis to directly investigate the effects of protein timing on strength and hypertrophic adaptations following long-term resistance training protocols. The study produced several novel findings. A simple pooled analysis of protein timing without controlling for covariates showed a significant effect on muscle hypertrophy (ES = 0.24 ± 0.10) with no significant effect found on muscle strength. It is generally accepted that an effect size of 0.2 is small, 0.5 is moderate, and 0.8 and above is a large, indicating that the effect of protein timing on gains in lean body mass were small to moderate. However, an expanded regression analysis found that any positive effects associated with protein timing on muscle protein accretion disappeared after controlling for covariates. Moreover, sub-analysis showed that discrepancies in total protein intake explained the majority of hypertrophic differences noted in timing studies. When taken together, these results would seem to refute the commonly held belief that the timing of protein intake in the immediate pre- and post-workout period is critical to muscular adaptations [3-5].

Perceived hypertrophic benefits seen in timing studies appear to be the result of an increased consumption of protein as opposed to temporal factors. In our reduced model, the amount of protein consumed was highly and significantly associated with hypertrophic gains. In fact, the reduced model revealed that total protein intake was by far the most important predictor of hypertrophy ES, with a ~0.2 increase in ES noted for every 0.5 g/kg increase in protein ingestion. While there is undoubtedly an upper threshold to this correlation, these findings underscore the importance of consuming higher amounts of protein when the goal is to maximize exercise-induced increases in muscle mass. Conversely, total protein intake did not have an impact on strength outcomes and ultimately was factored out during the model reduction process.

The Recommended Dietary Allowance (RDA) for protein is 0.8 g/kg/day. However, these values are based on the needs of sedentary individuals and are intended to represent a level of intake necessary to replace losses and hence avert deficiency; they do not reflect the requirements of hard training individuals seeking to increase lean mass. Studies do in fact show that those participating in intensive resistance training programs need significantly more protein to remain in a non-negative nitrogen balance. Position stands from multiple scientific bodies estimate these requirements to be approximately double that of the RDA [59,60]. Higher levels of protein consumption appear to be particularly important during the early stages of intense resistance training. Lemon et al. [61] displayed that novice bodybuilders required a protein intake of 1.6-1.7 g/kg/day to remain in a non-negative nitrogen balance. The increased protein requirements in novice subjects have been attributed to changes in muscle protein synthetic rate and the need to sustain greater lean mass rather than increased fuel utilization [62]. There is some evidence that protein requirements actually decrease slightly to approximately 1.4 g/kg/day in well-trained individuals because of a greater efficiency in dietary nitrogen utilization [63], although this hypothesis needs further study.

The average protein intake for controls in the unmatched studies was 1.33 g/kg/day while average intake for treatment was 1.66 g/kg/day. Since a preponderance of these studies involved untrained subjects, it seems probable that a majority of any gains in muscle mass would have been due to higher protein consumption by the treatment group. These findings are consistent with those of Cermak et al. [24], who found that protein supplementation alone produced beneficial adaptations when combined with resistance training. The study by Cermak et al. [24] did not evaluate any effects regarding timing of intake, however, so our results directly lend support to the theory that meeting target protein requirements is paramount with respect to exercise-induced muscle protein accretion; immediate intake of dietary protein pre and/or post-workout would at best appear to be a minor consideration. The findings also support previous recommendations that a protein consumption of at least 1.6 g/kg/day is necessary to maximize muscle protein accretion in individuals involved in resistance training programs [61].

For the matched studies, protein intake averaged 1.91 g/kg/day versus 1.81 g/kg/day for treatment and controls, respectively. This level of intake for both groups meets or exceeds suggested guidelines, allowing for a fair evaluation of temporal effects. Only 3 studies that employed matched protein intake met inclusion criteria for this analysis, however. Interestingly, 2 of the 3 showed no benefits from timing. Moreover, another matched study actually found significantly greater increases in strength and lean body mass from a time-divided protein dose (i.e. morning and evening) compared with the same dose provided around the resistance training session [19]. However, this study had to be excluded from our analysis because it lacked adequate data to calculate an ES. The sum results of the matched-protein studies suggest that timing is superfluous provided adequate protein is ingested, although the small number of studies limits the ability to draw firm conclusions on the matter.

This meta-analysis had a number of strengths. For one, the quality of studies evaluated was high, with an average PEDro score of 8.7. Also, the sample was relatively large (23 trials encompassing 478 subjects for strength outcomes and 525 subjects for hypertrophy outcomes), affording good statistical power. In addition, strict inclusion/exclusion criteria were employed to reduce the potential for bias. Combined, these factors provide good confidence in the ability draw relevant inferences from findings. Another strength was the rigid adherence to proper coding practices. Coding was carried out by two of the investigators (BJS and AAA) and then cross-checked between coders. Coder drift was then assessed by random selection of studies to further ensure consistency of data. Finally and importantly, the study benefited from the use of meta-regression. This afforded the ability to examine the impact of moderator variables on effect size and explain heterogenecity between studies [64]. Although initial findings indicated an advantage conferred by protein timing, meta-regression revealed that results were confounded by discrepancies in consumption. This ultimately led to the determination that total protein intake rather than temporal factors explained any perceived benefits.

There are several limitations to this analysis that should be taken into consideration when drawing evidence-based conclusions. First, timing of the meals in the control groups varied significantly from study to study. Some provided protein as soon as 2 hours post workout while others delayed consumption for many hours. A recent review by Aragon and Schoenfeld [23] postulated that the anabolic window of opportunity may be as long as 4–6 hours around a training session, depending on the size and composition of the meal. Because the timing of intake in controls were all treated similarly in this meta-analysis, it is difficult to determine whether a clear anabolic window exists for protein consumption beyond which muscular adaptations suffer.

Second, the majority of studies evaluated subjects who were inexperienced with resistance exercise. It is well-established that highly trained individuals respond differently to the demands of resistance training compared with those who lack training experience [65]. In part, this is attributed to a “ceiling effect” whereby gains in muscle mass become progressively more difficult as a trainee gets closer to his genetic hypertrophic potential. There also is emerging evidence showing that regimented resistance exercise attenuates anabolic intracellular signaling in rodents [66] and humans [67], conceivably diminishing the hypertrophic response. Our sub-analysis failed to show an interaction effect between resistance training status and protein timing for either strength or hypertrophy. However, statistical power was low because only 4 studies using trained subjects met inclusion criteria. Future research should therefore focus on determining the effects of protein timing on muscular adaptations in those with at least 1 year or more of regular, consistent resistance training experience.

Third, in an effort to keep our sample size sufficiently large, we pooled CSA and FFM data to determine hypertrophy ES. FFM is frequently used as a proxy for hypertrophy, as it is generally assumed that the vast majority of the gains in fat free mass from resistance training are myocellular in nature. Nevertheless, resistance exercise also is associated with the accretion of non-muscle tissue as well (i.e. bone, connective tissue, etc.). To account for any potential discrepancies in this regard, we performed sub-analyses on CSA and FFM alone and the results essentially did not change. For FFM, the difference between treatment and control was not significant (P = 0.27), with a ES difference of -0.08. Protein intake again was highly significant, with an ES impact of ~0.2 per every 1 g/kg/day. For CSA, the difference between treatment and control was not significant (P = 0.37), with a ES difference of -0.14. Protein intake was again significant (P = 0.02) with an ES impact of ~0.33 per every 0.5 g/kg.

Finally and importantly, there was a paucity of timing studies that attempted to match protein intake. As previously discussed, our results show that total protein intake is strongly and positively associated with post-exercise gains in muscle hypertrophy. Future studies should seek to control for this variable so that the true effects of timing, if any, can be accurately assessed.

### Practical applications

In conclusion, current evidence does not appear to support the claim that immediate (≤ 1 hour) consumption of protein pre- and/or post-workout significantly enhances strength- or hypertrophic-related adaptations to resistance exercise. The results of this meta-analysis indicate that if a peri-workout anabolic window of opportunity does in fact exist, the window for protein consumption would appear to be greater than one-hour before and after a resistance training session. Any positive effects noted in timing studies were found to be due to an increased protein intake rather than the temporal aspects of consumption, but a lack of matched studies makes it difficult to draw firm conclusions in this regard. The fact that protein consumption in non-supplemented subjects was below generally recommended intake for those involved in resistance training lends credence to this finding. Since causality cannot be directly drawn from our analysis, however, we must acknowledge the possibility that protein timing was in fact responsible for producing a positive effect and that the associated increase in protein intake is merely coincidental. Future research should seek to control for protein intake so that the true value regarding nutrient timing can be properly evaluated. Particular focus should be placed on carrying out these studies with well-trained subjects to better determine whether resistance training experience plays a role in the response.

## Competing interests

The authors declare that they have no competing interests.

## Authors’ contributions

BJS and AAA performed the literature search, performed quality assessment, and coded the studies. JWK devised and carried out the statistical analysis. All authors took part in writing the manuscript. All authors read and approved the final manuscript.
